# Mitochondrial Calcium Uniporter Deficiency in Zebrafish Causes Cardiomyopathy With Arrhythmia

**DOI:** 10.3389/fphys.2020.617492

**Published:** 2020-12-23

**Authors:** Adam D. Langenbacher, Hirohito Shimizu, Welkin Hsu, Yali Zhao, Alexandria Borges, Carla Koehler, Jau-Nian Chen

**Affiliations:** ^1^Department of Molecular, Cell and Developmental Biology, University of California, Los Angeles, Los Angeles, CA, United States; ^2^Department of Chemistry and Biochemistry, University of California, Los Angeles, Los Angeles, CA, United States

**Keywords:** mitochondria, cardiomyopathy, arrhythmia, mitochondrial calcium uptake, sinus arrest

## Abstract

Mitochondrial Ca^2 +^ uptake influences energy production, cell survival, and Ca^2 +^ signaling. The mitochondrial calcium uniporter, MCU, is the primary route for uptake of Ca^2 +^ into the mitochondrial matrix. We have generated a zebrafish *MCU* mutant that survives to adulthood and exhibits dramatic cardiac phenotypes resembling cardiomyopathy and sinus arrest. *MCU* hearts contract weakly and have a smaller ventricle with a thin compact layer and reduced trabecular density. Damaged myofibrils and swollen mitochondria were present in the ventricles of *MCU* mutants, along with gene expression changes indicative of cell stress and altered cardiac structure and function. Using electrocardiography, we found that *MCU* hearts display conduction system defects and abnormal rhythm, with extended pauses resembling episodes of sinus arrest. Together, our findings suggest that proper mitochondrial Ca^2 +^ homeostasis is crucial for maintaining a healthy adult heart, and establish the *MCU* mutant as a useful model for understanding the role of mitochondrial Ca^2 +^ handling in adult cardiac biology.

## Introduction

Calcium flux through the plasma membrane and intracellular organelles regulates cardiac contraction and energy metabolism. During the cardiac cycle, Ca^2 +^ released from the sarcoplasmic reticulum initiates cardiac contraction and the entry of Ca^2 +^ into the mitochondrial matrix activates critical enzymes of the TCA cycle and electron transport chain to promote ATP production. In addition to linking excitation-contraction coupling to energy metabolism, mitochondrial Ca^2 +^ uptake also shapes Ca^2 +^ signals and regulates cell survival.

The mitochondrial calcium uniporter (MCU) is a transmembrane channel protein located on the inner mitochondrial membrane that is capable of transporting Ca^2 +^ into the mitochondrial matrix. Genetic manipulations *in vitro* and *in vivo* have established MCU’s critical roles in the heart. Manipulating MCU expression alters Ca^2 +^ transients in neonatal cardiomyocytes (Drago et al., 2012) and myocardial MCU inhibition impairs physiological fight-or-flight responses (Wu et al., 2015). Furthermore, ablation of MCU activity in cardiomyocytes abolishes mitochondrial Ca^2 +^ uptake, dampens acute cardiac responses to ß-adrenergic receptor stimulation, and exerts a cardioprotective effect in an *in vivo* ischemia-reperfusion injury model by preventing the activation of the mitochondrial permeability transition pore (Kwong et al., 2015; Luongo et al., 2015). Given the important physiological roles of mitochondrial Ca^2 +^ uptake in the heart, it is surprising that mice lacking MCU activity in cardiomyocytes have normal baseline cardiac function, TCA cycle and electron transport chain activity, and ATP production (Kwong et al., 2015; Luongo et al., 2015). This unexpected phenotype may be a result of strong compensatory mechanisms, as impaired physiological intracellular Ca^2 +^ homeostasis and altered expression of Ca^2 +^ flux-regulatory genes were noted when MCU is inhibited (Kwong et al., 2015; Rasmussen et al., 2015). Additional MCU-deficient animal models will assist in the investigation of the cardiac physiological roles of MCU.

The zebrafish model is highly amenable to genetic manipulations and has a primitive vertebrate heart, consisting of one ventricle and one atrium, with physiology that is governed by the same molecular and cellular mechanisms that control the human heart. Like all vertebrate hearts, zebrafish cardiac contraction is strongly influenced by Ca^2 +^ flux. Manipulating Ca^2 +^ flux regulatory genes often results in early embryonic contractile and rhythm dysfunction. For example, Ca^2 +^ extrusion from cardiomyocytes is primarily carried out by the Sodium Calcium Exchanger NCX1. In zebrafish, the *tremblor* locus encodes *ncx1h/slc8a1a*, a cardiac-specific form of NCX1 (Ebert et al., 2005; Langenbacher et al., 2005). Loss of *ncx1h/slc8a1a* results in Ca^2 +^ overload and a fibrillation-like cardiac defect (Ebert et al., 2005; Langenbacher et al., 2005) along with deterioration of myofibrils (Shimizu et al., 2017). Interestingly, promoting Ca^2 +^ uptake into mitochondria by potentiation of the mitochondrial outer membrane channel VDAC or overexpression of MCU can restore rhythmic cardiac contractions to *ncx1h/slc8a1a*-deficient embryos (Shimizu et al., 2015), suggesting that mitochondria are capable of serving as a buffer for elevated cytosolic Ca^2 +^ and indicating that MCU is functional in cardiomyocytes at a very early developmental stage.

How MCU deficiency impacts cardiac function in zebrafish is not known. In this study, we employed the TALEN genome editing approach to create a null allele of MCU. We show that adult *MCU* mutants exhibit cardiac remodeling resembling cardiomyopathy providing an animal model to assess MCU’s physiological roles in the heart.

## Results

### Generation of an *MCU* Mutant

**FIGURE 1:**
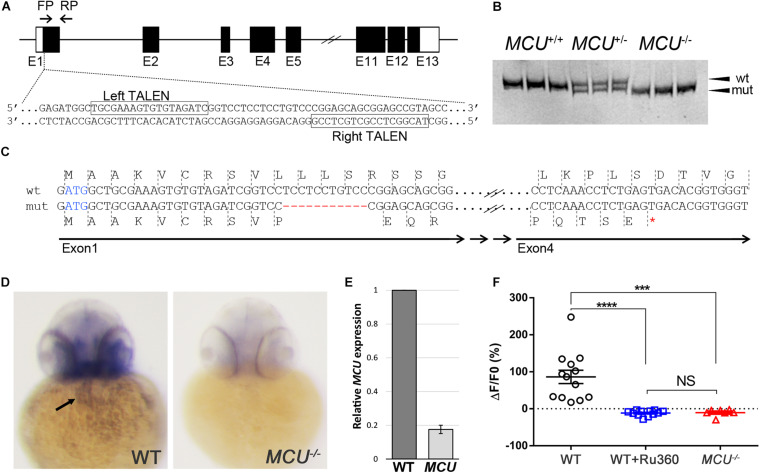
Targeted mutation of *MCU* using TALENs. **(A)** Schematic representation of partial *MCU* genomic sequence demonstrating the TALEN binding sites. Open and solid boxes represent UTRs and coding exons, respectively. **(B)** Representative genotyping results. Amplification of the genomic region surrounding the deletion using a primer pair FP/RP shown in **(A)**. The wildtype (152 bp) and mutant (141 bp) fragments were separated using polyacrylamide gel electrophoresis. **(C)** Sequencing analysis of cDNA identified a frameshift mutation caused by an 11-nucleotide deletion in exon 1, resulting in a premature termination of translation. **(D)** *In situ* hybridization detects *MCU* transcripts in the heart (arrow) in WT but not *mcu* mutant embryos at 3 days post fertilization (dpf). **(E)** Quantitative PCR analysis of *MCU* transcripts in 3 dpf embryos. **(F)** Quantification of Rhod 2 Signals in WT cardiomyocytes with and without MCU inhibitor Ru360 and *MCU* mutant cardiomyocytes. ****p < 0.0001; ***p < 0.001; and NS: *p* > 0.05.

To investigate the impacts of *MCU* deficiency on cardiac structure and function in the zebrafish, we employed the TALEN genome editing approach to target the *MCU* locus (Figure 1A). We identified *MCU^*LA*2446^*, an allele that carries an 11-nucleotide deletion within the first exon of *MCU* (Figures 1A,B). This internal deletion produces a frameshift after the 10th residue and a premature stop codon, resulting in a mutant protein that lacks the majority the MCU protein’s sequence including its conserved coil-coil and transmembrane domains (Figure 1C).

Whole mount *in situ* hybridization shows that *MCU* is expressed in a wide range of tissues including the heart (Figure 1D; Shimizu et al., 2015). Interestingly, the expression of *MCU* was not detectable in *MCU^*LA*2446^* homozygous embryos (8/8 wild type embryos with normal expression vs. 7/7 *MCU^*LA*2446^* homozygous embryos with no expression), suggesting that *MCU^*LA*2446^* mutant mRNA undergoes degradation, likely through nonsense-mediated decay (Figure 1D). Supporting this notion, we also observed an 82% reduction in *MCU* transcripts in *MCU* mutant embryos by quantitative real-time PCR (Figure 1E).

As the primary conduit facilitating the entry of Ca^2 +^ into the mitochondrial matrix, loss of MCU is predicted to attenuate mitochondrial Ca^2 +^ uptake. Indeed, mitochondrial Ca^2 +^ levels were elevated upon induction in wild type (WT) cardiomyocytes, but this mitochondrial Ca^2 +^ uptake is suppressed by treatment with the MCU inhibitor Ru360 and is absent in *MCU^*LA*2446^* homozygous cardiomyocytes (Figure 1F). Collectively, the unstable transcript, the predicted truncated protein sequence and the severe mitochondrial Ca^2 +^ uptake defect indicate that *MCU^*LA*2446^* is likely to be a null allele.

### The *MCU* Knockout Is Homozygous Viable in Zebrafish

**FIGURE 2:**
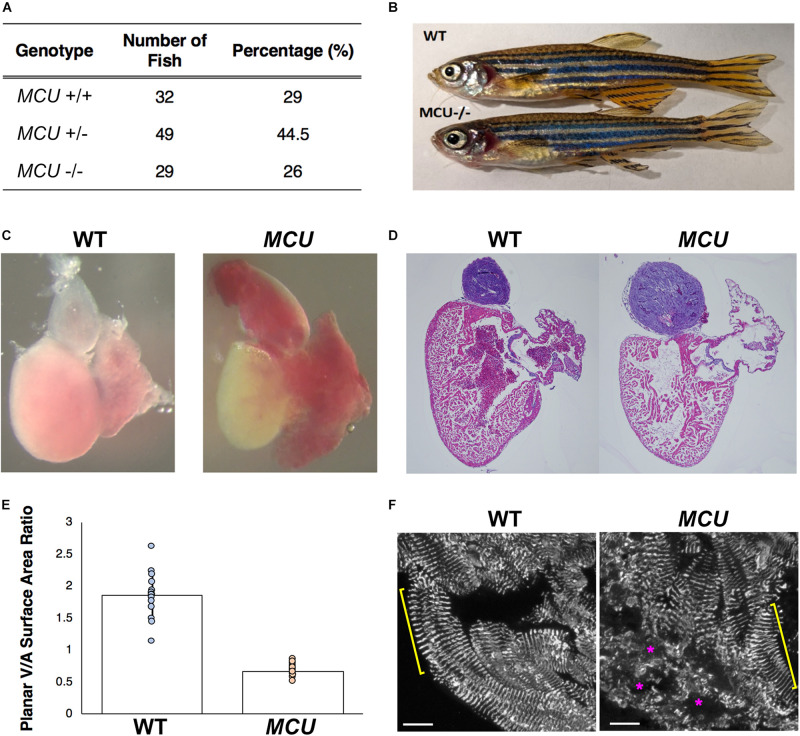
Cardiac morphological analysis of adult *MCU* mutants. **(A)** Genotypic distribution of F1 generation from *MCU* heterozygous parents matches closely with Mendelian ratio and show no signs of embryonic lethality. **(B)** Comparable exterior phenotypic traits between wildtype and *MCU* mutant zebrafish. **(C)** Defects in cardiac structure observed in dissected adult *MCU* mutant hearts. Mutant hearts were characterized by enlarged atrial volume and limited ventricular blood flow. **(D)** Reduction in tissue density and disorganization of myocardium observed in histological sections (Hematoxylin and Eosin stains) of *MCU* mutant hearts. Nuclei rich regions stained blue and cytoplasmic regions and ECM stained pink. **(E)** Selected image frames during systolic and diastolic phases were analyzed to compare ventricular-to-atrial surface area ratio. Ventricular:atrial SA ratio were significantly reduced in *MCU* mutant hearts (*n* = 14 for each group, *p* < 0.0001). **(F)** α-actinin staining marks the *Z*-lines of intact sarcomeres in ventricular cardiomyocytes of wild type adult fish (yellow bracket). Adult *MCU* ventricles contain regions with intact myofibrils/sarcomeres (yellow bracket), but also exhibit areas of patchy α-actinin staining indicative of damaged myofibrils and broken-down sarcomeres (magenta asterisks). Scale bars indicate 10 μm.

Following Mendel’s Law, approximately 25% of embryos collected from crosses of *MCU^*LA*2446^* heterozygotes are homozygous for the *MCU^*LA*2446^* allele. These *MCU^*LA*2446^* homozygous mutant embryos are phenotypically indistinguishable from their siblings throughout the embryonic stage (1 to 5 days post fertilization). We next asked whether the *MCU* mutant fish could survive to adulthood. We genotyped 110 4-month-old fish raised from *MCU^*LA*2446^* heterozygous crosses. An approximately 1:2:1 ratio among *MCU*+/+, +/- and -/- individuals was noted in this population (Figure 2A). The *MCU* mutant fish are of normal size (Figure 2B) and have similar a lifespan to their WT siblings, demonstrating that loss of *MCU* does not compromise the growth or viability of the fish.

### MCU Deficiency Results in Cardiomyopathy in Zebrafish

Mitochondrial dysfunction often induces cardiac remodeling and impairs cardiac function. We examined whether *MCU^*LA*2446^* mutants manifest cardiac defects and found that the embryonic *MCU* mutant hearts have normal morphology and function (not shown). However, all adult *MCU^*LA*2446^* mutant hearts exhibit phenotypes resembling cardiomyopathy. In sharp contrast to the well-structured cardiac chambers of the WT zebrafish heart, the *MCU* mutant heart is dysmorphic with a thin, dilated atrium and a small ventricle (Figures 2C,D). To quantify the relative sizes of the cardiac chambers, we measured the surface areas of the ventricle and atrium and found that the ratio of ventricular surface area (VSA) to atrial surface area (ASA) is reduced by 3.6-fold in *MCU* mutants compared to WT (VSA/ASA = 1.865 in the controls and 0.668 in *MCU* mutants, *n* = 14 for each group, *p* < 0.0001; Figure 2E).

**FIGURE 3:**
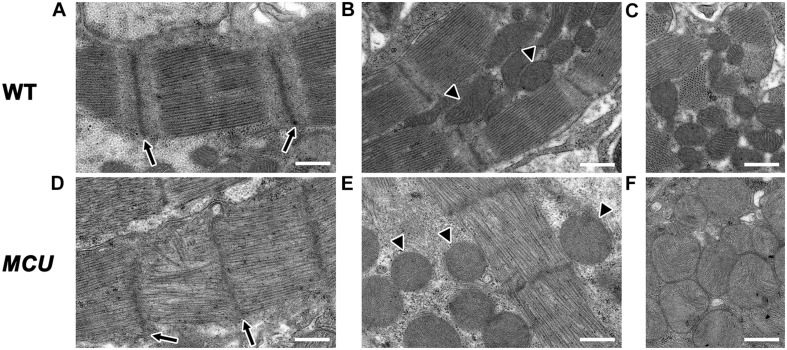
Transmission electron microscopy analysis of *MCU* mutant ventricles. Representative TEM images of WT **(A–C)** and *MCU* **(D–F)** ventricular cardiomyocytes. **(A,D)** WT sarcomeres display clear *Z*-lines (arrows in **A**) separated by parallel bands of myosin thick filaments. *MCU Z*-lines are less well defined and patchy (arrows in **D**) and thick filaments are sometimes disorganized. **(B,C)** WT myofibrils are closely associated with oval-shaped mitochondria containing tightly packed cristae (arrowheads in **B** mark examples, **C** shows more examples). **(E,F)** *MCU* myofibrils are also closely associated with mitochondria, but they are less electron-dense and display a round and swollen morphology with more loosely packed cristae [arrowheads in **(E)** mark examples, **(F)** shows more examples]. Scale bars indicate 500 nm.

The relative reduction in the size of the ventricle in *MCU* was also accompanied by numerous signs of chamber remodeling and cellular deterioration. The compact layer in *MCU* ventricles was thinner than in WT control ventricles, and the density of trabecula was reduced (Figure 2D), indicating a severe reduction in the amount of contractile tissue in the *MCU* heart. Additionally, patches of disrupted α-actinin protein localization were present in *MCU* ventricular cardiomyocytes (average of 20% of observed area with patchy α-actinin staining in *MCU* mutants, *n* = 3 vs. no patchy α-actinin staining in WT, *n* = 3, *p* = 0.058), suggesting the presence of disassembled sarcomeres (Figure 2F). We used transmission electron microscopy (TEM) to further explore the cellular changes in *MCU* and found that *MCU* ventricular cardiomyocytes exhibit very abnormal mitochondria and myofibril structure. WT cardiomyocytes have myofibrils with well-defined *Z*-lines and organized thick filaments (Figures 3A,B). These myofibrils closely associate with many oval-shaped mitochondria containing tightly packed cristae (Figures 3B,C). *MCU* myofibrils, on the other hand, sometimes have patchy *Z*-lines and disoriented thick filaments that are not organized in a parallel fashion (Figures 3D,E). While mitochondria were abundant in *MCU* ventricular cardiomyocytes and associated with myofibrils, they displayed a round, swollen morphology with loosely packed cristae (Figures 3E,F). These data suggest that in addition to the reduction in the relative size of the ventricle of *MCU*, the remaining ventricular cardiomyocytes exhibit mitochondrial stress and myofibril damage.

### Impaired Cardiac Function of *MCU* Mutants

**FIGURE 4:**
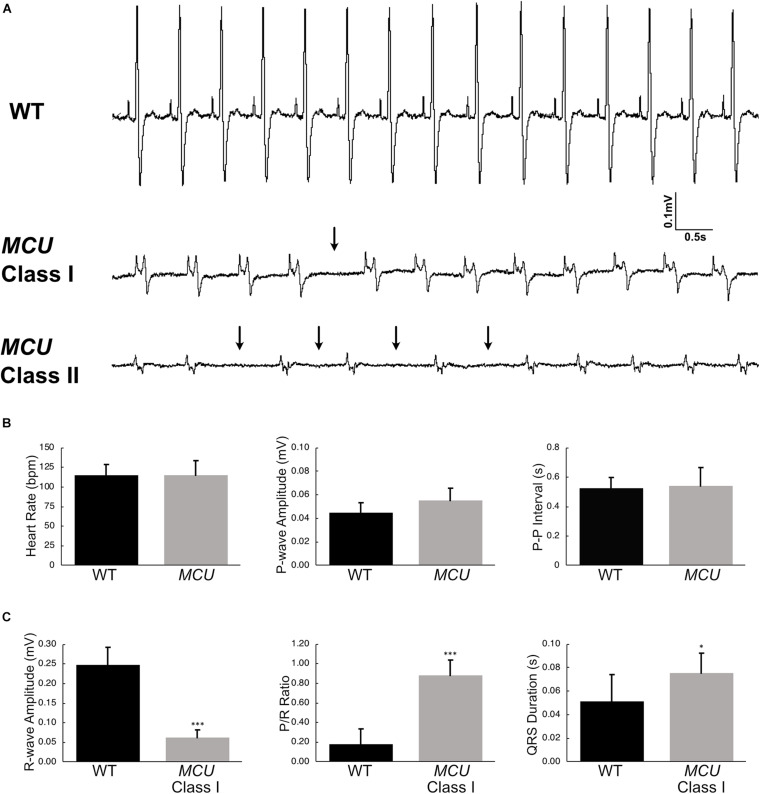
Electrocardiogram analysis of wildtype and *MCU* mutant fish. **(A)** ECG tracings of wildtype fish (top), Class I *MCU* mutant (clearly distinguishable QRS complex, middle) and Class II *MCU* mutant (highly abnormal QRS complex, bottom) highlight episodes of long beat-to-beat pauses. Arrows point to beat intervals that are greater than mean + 2σ. **(B)** Analysis and comparison of ECG indices between wildtype and *MCU* mutants (Class I and II combined). No significant differences were identified in heart rate (115.4 bpm v 115.0 bpm, *p* > 0.05), *P*-amplitude (0.045 mV v 0.055 mV, *p* > 0.05), and *P*-*P* interval (0.524 s v 0.539 s, *p* > 0.05) between wildtype and mutant, respectively. **(C)** Statistical differences were present in *R*-wave amplitudes (reduced in *MCU* Class I mutant, 0.248 mV v 0.062 mV, *p* < 0.0001), *R*-wave amplitude-to-*P*-wave amplitude ratio (increased in *MCU* Class I mutant, 0.182 v 0.884, *p* < 0.001), and duration of the QRS complex (prolonged in *MCU* Class I mutant, 0.0512 s v 0.0721 s, *p* < 0.05). *p < 0.05; ***p < 0.0001.

Explanted WT zebrafish hearts exert strong rhythmic contractions (Supplementary Video 1). *MCU* mutant atria contract weakly and contraction of the ventricle is difficult to observe (Supplementary Video 2), indicating that *MCU* loss compromises cardiac function. To assess cardiac performance *in vivo*, we acquired electrocardiogram (ECG) signals from WT and *MCU* mutant fish aged between 7 to 10 months (Figure 4A). In line with previous reports (Yan et al., 2020), we measured an average WT heart rate of 115 ± 14 beats per minute (bpm) at ambient temperature with little beat-to-beat variability (standard deviation of *P*-*P* interval: 0.074 ± 0.017 s, *n* = 14; Figure 4B).

We noted multiple abnormalities in the ECG signals from *MCU* mutant hearts and realized that *MCU* can be categorized into two groups based on the appearance of their ECG waveforms. In one group of *MCU* mutants (9 out of 14 *MCU* mutants analyzed, Class I), ECG signals consist of clearly distinguishable *P* waves and QRS complexes (Figure 4A, Class I). The other group of *MCU* mutants (5 out 14, Class II) have highly abnormal or indistinguishable QRS complexes (Figure 4A, Class II). Overall, the average heart rate and P-wave amplitude of *MCU* mutants was similar to that observed in controls, but variability of the beat-to-beat interval was significantly increased (standard deviation of *P*-*P* interval: 0.135 ± 0.031 s, *n* = 14, and *p* < 0.01; Figure 4B). Class II *MCU* mutants displayed an even greater increase in the variability of the beat-to-beat interval compared to Class I mutants (standard deviation of *P*-*P* interval for Class I *MCU* mutants: 0.103, *n* = 9, standard deviation of *P*-*P* interval for Class II *MCU* mutants: 0.148, *n* = 5, and *p* < 0.05).

Since *MCU* Class II mutants lack a reliably distinguishable QRS complex, we examined ECG parameters related to the *R*-wave in Class I mutants only. In *MCU* Class I mutants, the maximum amplitude of the *R* wave was reduced by approximately 5-fold compared to WT (0.248 mV in WT vs 0.0626 mV in Class I mutants, *n* = 14 for WT, *n* = 9 for Class I mutants, *p* < 0.0001) and concomitantly the P/R Ratio was significantly increased (Figure 4C), reflecting the decreased size and strength of the *MCU* ventricle. Interestingly, we also noted that the PR interval was increased by 50% (0.033 s vs. 0.050 s, for WT and Class I mutants, respectively, *p* < 0.01) and the QRS duration was increased by 40% in *MCU* Class I mutants compared to WT (0.0512 ms vs. 0.0721 ms, for WT and Class I mutants, respectively, *p* < 0.05; Figure 4C), suggesting conduction problems at the atrioventricular node and within the ventricular myocardium. Together, the abnormalities present in *MCU* ECGs suggest a weakened ventricle with abnormalities in the conduction of electrical impulses.

### Increased Incidence of Sinoatrial Arrest in *MCU* Mutants

**FIGURE 5:**
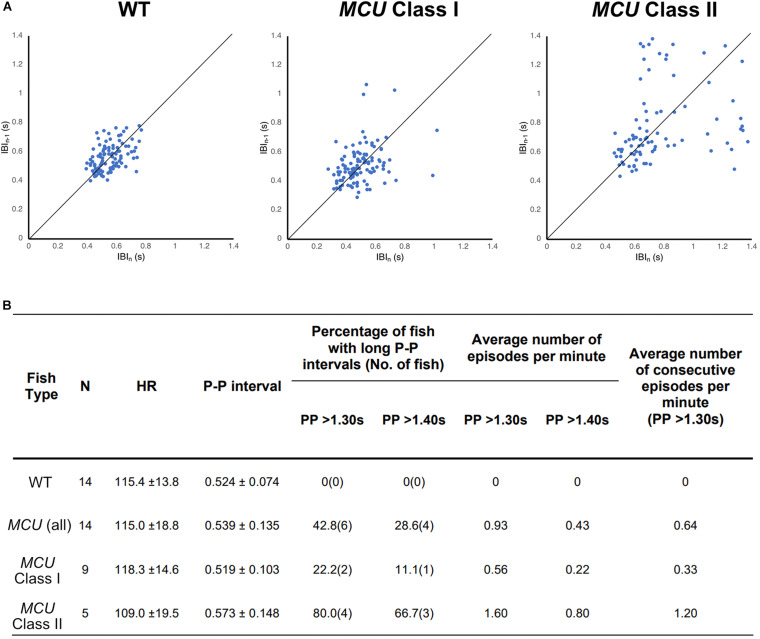
Analysis on cardiac arrhythmia in *MCU* mutant. **(A)** Increased heartbeat variability in *MCU* mutants displayed via Poincaré plots of consecutively occurring *P*-*P* intervals in adult wildtype and MCU mutant fish. **(B)** Summary of ECG data from 14 wildtype fish and 14 *MCU* mutant fish. Different cut-offs of *P*-*P* intervals were used to classify severity and definitive of occurring sinoatrial block episodes. Increased sample percentage with SA block episodes, per minute frequency, and consecutively occurring episodes were identified in *MCU* mutant (compared to wildtype), and further emphasized in *MCU* Class II mutants.

To visualize the regularity of heartbeats in WT and *MCU* mutant hearts, we plotted consecutive interbeat intervals (IBI) of WT and *MCU* mutants. While most WT IBIs clustered around the identity line (IBI_n__–__1_ = IBI_n_), suggesting normal cardiac rhythm, the consecutive IBIs in *MCU*, and in particular Class II mutants, were highly variable with frequent long pauses (Figure 5A), suggesting that *MCU* deficiency may cause sinoatrial node dysfunction.

Sinoatrial node dysfunction often results in sinus arrest (SA), in which the sinoatrial node temporarily fails to discharge an impulse. In humans, the resting heart rate ranges from 60 to 100 bpm and a 2 s beat-to-beat interval is considered a definitive SA episode. In our dataset, the heart rates of wild type fish ranged between 110–120 bpm with an average beat-to-beat interval of 0.52 s. The beat-to-beat intervals in wild type fish were typically very close to the average, with no wild type fish displaying a beat-to-beat interval of greater than 1.3 s (a 2.5-fold increase compared to normal) within a 1-min recording period (*n* = 14, 1823 beats analyzed; Figure 5B). We therefore chose a 1.3 s interval as the cutoff for a bone fide SA episode in this study. Strikingly, while their average beat-to-beat intervals were not significantly different from WT (*p* > 0.05), *MCU* mutants frequently manifested episodes of SA, with six out of 14 *MCU* mutants exhibiting SA within the 1-min ECG recording period (1740 beats analyzed, *p* < 0.001; Figure 5B). Within the *MCU* mutant population, the incidences of SA episodes varied between the two phenotypic classes we identified (*p* < 0.05). 22% of Class I *MCU* mutants displayed SA with a frequency of 0.56 episodes per minute (1096 beats analyzed) whereas 80% of Class II *MCU* mutants displayed SA (1.60 episodes per minute, 644 beats analyzed; Figure 5B). Incidences of consecutively occurring SA episodes (a minimum of two consecutive > 1.3 s pauses) were also observed in *MCU* mutants (0.64 consecutive episodes per minute) with significantly more frequent occurrence in Class II mutants (1.20 consecutive episode per minute, *p* < 0.05). Together, these findings indicate that *MCU* deficiency increases SA susceptibility.

### Hearts of *MCU* Mutant Fish Display Gene Expression Changes Reflecting Altered Cardiac Structure and Function

**FIGURE 6:**
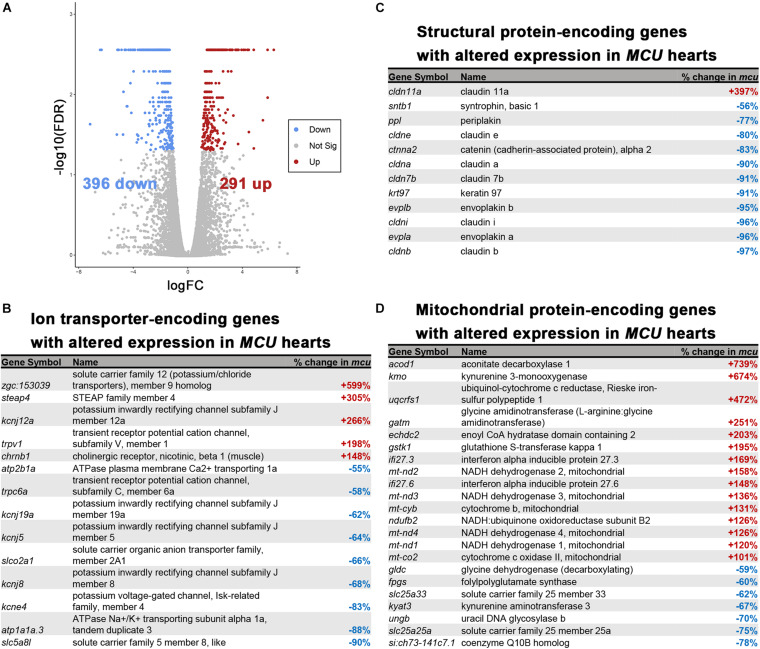
RNA-seq analysis of the *MCU* mutant adult heart. **(A)** Volcano plot of differential gene expression of *MCU* mutant hearts vs. wildtype. Each point represents the average value of one transcript in duplicate experiments. The expression difference is considered significant for an adjusted *p*-value (FDR) of less than 0.05. Red points represent genes significantly upregulated in *MCU* mutants and blue points represent significantly down regulated genes. FDR, false-discovery rate. **(B–D)** Selected lists of significantly differentially expressed (FDR < 0.05) ion transporter-encoding **(B)**, structural protein-encoding **(C)**, and mitochondrial protein-encoding **(D)** genes. The percent increase (+, red) or decrease (-, blue) in expression in *MCU* compared to WT, based on the fold-changes determined by Cufflinks, is displayed in the right column.

Cardiac remodeling and dysfunction are often associated with transcriptomic alteration. To better understand the transcriptomic responses to loss of MCU activity, we performed RNA-seq on adult 7-month old WT and *MCU* mutant hearts. We detected 687 genes that were differentially expressed between WT and *MCU* mutants (false discovery rate = 0.05) of which 291 gene were upregulated in *MCU* and 396 genes were downregulated (Figure 6A). Many genes relevant to cardiac biology exhibited significantly altered expression in *MCU* hearts (Figures 6B–D). Consistent with the abnormal function of *MCU* hearts, a large number of ion transporter-encoding genes were dysregulated, including 7 channels with predicted potassium transporting activity (*zgc:153039*, *kcnj12a*, *kcnj19a*, *kcnj5*, *kcnj8*, *kcne4*, and *atp1a1a.3*) and a homolog of the plasma membrane Ca^2 +^ transporter PMCA1 (*atp2b1a*; Figure 6B and Supplementary Figure 1). We also found that putative structural components of cell junctions (tight junctions: *cldn11a*, *cldne*, *cldna*, *cldn7b*, *cldni*, and *cldnb*; adherens junctions: *ctnna*; and desmosomes: *ppl*, *evplb*, and *evpla*) showed significantly altered expression in *MCU* hearts (Figure 6C and Supplementary Figure 1), suggesting that the communication between and integrity of cardiac cells in *MCU* may be compromised. Additionally, we noted that in line with the mitochondrial dysfunction expected upon loss of *MCU* activity, 22 mitochondrial protein-encoding genes were differentially expressed between wildtype and *MCU* hearts (Figure 6D). Interestingly 6 mitochondrial genome-encoded members of the electron transport chain (*mt-nd2*, *mt-nd3*, *mt-cyb*, *mt-nd4*, *mt-nd1*, and *mt-co2*) were all upregulated in *MCU* (Figure 6D). To gain a deeper understanding of the gene networks that were dysregulated in *MCU* hearts, we tested the sets of genes that were up- or downregulated for gene ontology over-representation.

**FIGURE 7:**
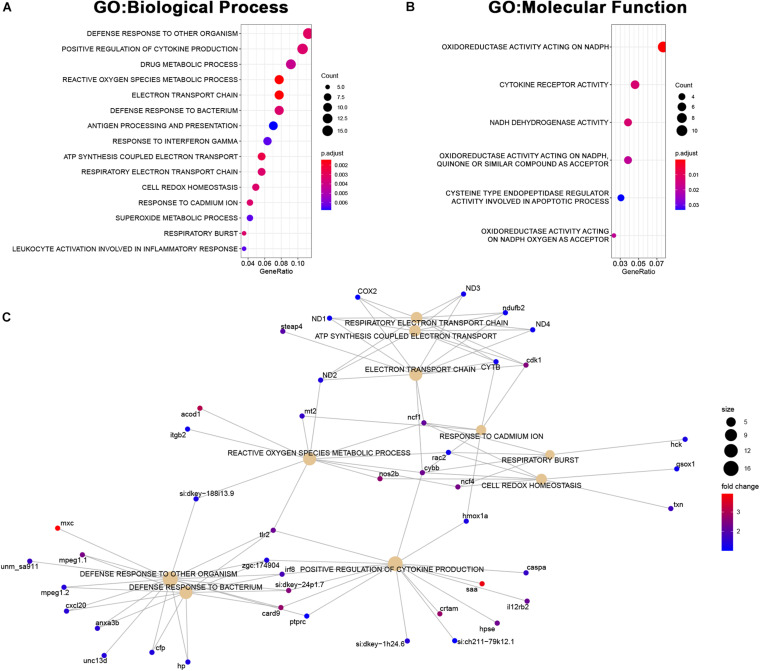
Upregulated genes in *MCU* hearts reflect altered mitochondrial biology. **(A, B)** Dot plots of over-represented Biological Process **(A)** and Molecular Function **(B)** gene ontology terms in the set of genes that is significantly upregulated in *MCU* mutant hearts. The dot size indicates the number of genes associated with each term and the dot color indicates the significance of the enrichment (adjusted *p*-values). **(C)** Gene concept networks of the top 10 over-represented Biological Process gene ontology terms in the set of genes that is significantly upregulated in *MCU* mutants. The dot size indicates the number of genes associated with each term and dots representing genes are colored based on their fold-change [log2(fold-change)].

The set of genes upregulated in *MCU* was highly enriched with genes involved in inflammatory response (defense response to other organisms, positive regulation of cytokine production) and mitochondrial function (reactive oxygen species metabolic process, electron transport chain, ATP synthesis coupled electron transport; Figures 7A–C) indicating that oxidative stress and compensatory pathways are activated in response to the altered mitochondrial function in *MCU* mutant hearts. Strikingly, three key mediators of ROS production (*nos2b*, *cybb*, and *ncf4*) were greater than 4-fold upregulated upon loss of MCU activity (Figure 7C). Increased ROS production is both a cause of and response to mitochondrial dysfunction (Bhatti et al., 2017) and promotes the release of damaging pro-inflammatory cytokines (Suematsu et al., 2003; Bulua et al., 2011), suggesting that in the adult zebrafish heart, MCU plays an essential role in maintaining healthy mitochondria.

**FIGURE 8:**
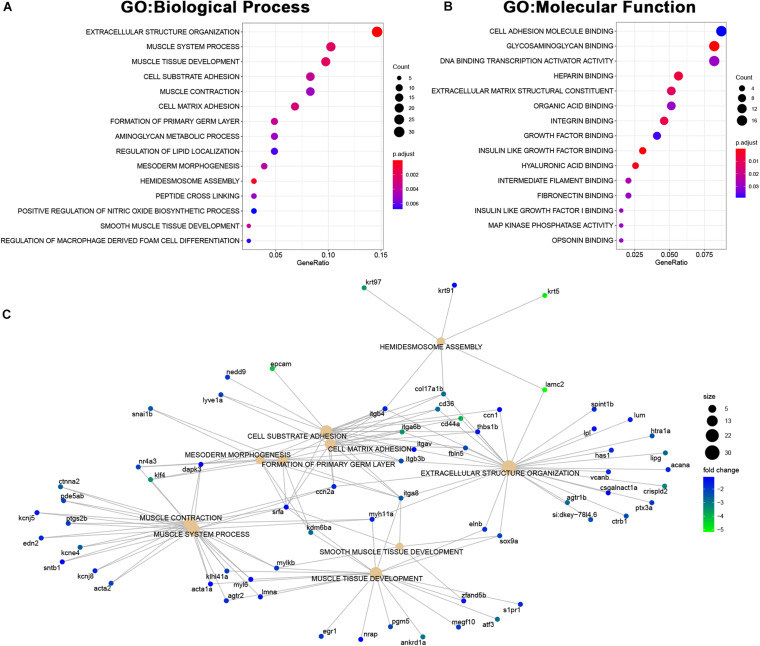
Downregulated genes in *MCU* hearts suggest defects in cardiac structure and function. **(A,B)** Dot plots of over-represented Biological Process **(A)** and Molecular Function **(B)** gene ontology terms in the set of genes that is significantly downregulated in *MCU* mutant hearts. The dot size indicates the number of genes associated with each term and the dot color indicates the significance of the enrichment (adjusted *p*-values). **(C)** Gene concept networks of the top 10 over-represented Biological Process gene ontology terms in the set of genes that is significantly downregulated in *MCU* mutants. The dot size indicates the number of genes associated with each term and dots representing genes are colored based on their fold-change [log2(fold-change)].

Among the genes downregulated in *MCU* mutant hearts, we found a significant enrichment of genes involved in both cardiac structure (extracellular structure organization, muscle tissue development, cell substrate adhesion, mesoderm morphogenesis, hemidesmosome assembly, cell adhesion molecule binding, and extracellular matrix structural component) and function (muscle system process, muscle contraction; Figures 8A–C). Complementary to the changes in structural protein expression we identified (Figure 6C), we noted a decrease in the expression of genes involved in proteoglycan deposition (*acana*, *vcanb*, and *has1*) and the formation and maintenance of desmosome junctions (*itgb4*, *col17a1b*, and *lamc2*) in *MCU* hearts (Figure 8C). Cumulatively, these structural gene expression changes may contribute to the damaged myofibrils and disassembled sarcomeres we observed in the *MCU* ventricle (Figure 2F). Intriguingly, we also noticed that two inward-rectifier type potassium channels (*kcnj5*, *kcnj8*) associated with the gene ontology term “muscle contraction” were greater than 2-fold downregulated in *MCU* hearts. Consistent with the SA node defects we observed in our EKG analysis, variations in *KCNJ5* have been linked to sinus node dysfunction in human patients (Holmegard et al., 2010; Kuss et al., 2019; Yamada et al., 2019), and mutation of *KCNJ8* in the mouse model results in episodes of sinus arrest (Aziz et al., 2018).

## Discussion

In this study, we described the pathophysiological changes to the heart resulting from loss of function of *MCU* in zebrafish. The *MCU^*LA*2446^* knockout allele results in a frameshift after the 10th amino acid of the MCU protein and an early stop codon, causing nonsense mediated decay of the mutant transcripts and suggesting that *MCU^*LA*2446^* is a true null allele. Interestingly, while MCU activity appears to be dispensable for embryonic development in zebrafish, adult *MCU* mutants exhibited cardiac remodeling resembling cardiomyopathy along with functional defects including sinus arrest.

Although *MCU* mutants survive to adulthood at a rate that is similar to their wild type siblings, their hearts display marked structural abnormalities. Both chambers exhibit a sparse myocardial tissue distribution, and deteriorating myofibrils and swollen mitochondria were present in the ventricles of *MCU* hearts. In particular, the ventricles of *MCU* hearts were proportionately smaller than those of wild type fish. Our transcriptomic analysis of *MCU* hearts revealed that in line with the swollen mitochondrial morphology we observed, ROS production and stress pathways were significantly upregulated in *MCU* hearts. Simultaneously, proteins important for muscle contraction, ECM adhesion and deposition, and cell-cell junctions were significantly downregulated. These transcriptomic changes suggest that mitochondrial dysfunction produced by loss of MCU activity leads to increased cell stress, a loss of tissue integrity, and reduced cell-cell communication, resulting in a pathological remodeling process.

Consequently, we observed functional defects in *MCU* hearts using electrocardiograph analysis. Notably, a dramatic decrease in R-wave amplitudes, indicative of severely impaired ventricular contraction, was present in *MCU* mutants. Additionally, we observed signs of slowed ventricular depolarization and conduction system defects in the atrioventricular node and ventricular myocardium of *MCU* hearts. These abnormalities were consistent with the decreased contractile tissue present in *MCU* ventricles, and may also reflect the changes in cell-cell junctional communication and ion channel expression revealed by our transcriptomic analysis. Additionally, we found that *MCU* mutants can be divided into 2 classes of functional severity based on the presence of a distinguishable QRS complex during ECG examination. Intriguingly, Class II mutants exhibited a higher incidence of SA and general increase in variability of the beat-to-beat interval compared to Class I mutants. The absence of a clearly distinguishable QRS complex in this subgroup of *MCU* mutants demonstrates that they have even more severe ventricular functional defects than are present in Class I mutants. These findings suggest that the accumulation of cellular stress and myofibril damage in *MCU* may induce progressive changes in adult cardiac physiology including damage to cardiomyocyte structure and the cardiac conduction system.

Limited animal models have been developed to elucidate the role of mitochondrial calcium influx in cardiac arrhythmia and cardiomyopathies. In mice, compensatory mechanisms appear to exist that preserve adult cardiac function when mitochondrial Ca^2 +^ influx is perturbed. Knockout of MCU activity in the adult mouse heart is accompanied by a corresponding decrease in the protein levels of the mitochondrial sodium calcium exchanger NCLX, a major mitochondrial calcium efflux mediator, potentially explaining why MCU KO hearts display normal baseline function (Kwong et al., 2015). Interestingly, MCUKO ventricular myocytes also exhibit an increased sodium calcium exchanger (NCX) current, suggesting that extramitochondrial changes in plasma membrane calcium extrusion may also be involved in maintaining normal cardiac physiology in the absence of MCU (Rasmussen et al., 2015). We did not detect changes in the expression of a broad range of Ca^2 +^ transporters and ion channels in *MCU* mutants, including L-type Ca^2 +^ channels (*cacna1sa*, *cacna1sb*, *cacna1c*, *cacna1da*, and *cacna1db*), HCN4 (*hcn4*, *hcn4l*), SCN5A (*scn12aa*, *scn5lab*), SERCA (*atp2a2a*), RYR2 (*ryr2a*, *ryr2b*), NCX1 (*slc8a1a*), VDAC (*vdac1*, *vdac2*, and *vdac3*), or other components of the MCU complex (*mcur1*, *micu1*, *micu2*, *micu3a*, *micu3b*, and *smdt1a*), suggesting that widespread compensatory changes may not be occurring upon loss of MCU activity in zebrafish. Further studies will be necessary to determine if any subtle compensatory changes occur in zebrafish hearts lacking MCU activity, and to reveal the molecular and cellular mechanisms underpinning the changes in cardiac structure and function that occur between embryonic development and the adult disease state that we focused on in this study. Given the complex interplay between heart function and morphology, it is possible that MCU activity within the contractile myocardium, conduction system, other cell types, or a combination of cell types is needed for normal growth and maintenance of the heart. For example, loss of MCU may result in diminished expansion of the myocardium during juvenile stages when the heart is growing as well as increased cell death in the mature myocardium. The zebrafish *MCU* mutant is therefore a novel model offering opportunities to study the progressive deterioration in cardiac structure and function, from embryogenesis to adulthood, that is caused by defective mitochondrial Ca^2 +^ uptake.

In summary, our findings shed light on the role of mitochondrial calcium homeostasis by exploring cardiac morphological and functional defects in *MCU* mutants. Proper mitochondrial calcium influx is required for normal cardiac structural development and long-term function. The loss of MCU results in cumulative pathological effects that are progressively manifested as mutants approach adulthood. Critically, characteristics of ECG tracings of mutants, including the presence of arrhythmicity, could be of significance in understanding the involvement of calcium signaling and homeostasis in heart disease. Future studies on related pathways are needed to clarify the role of mitochondrial calcium homeostasis in proper cardiac function.

## Materials and Methods

### Fish Husbandry

Zebrafish were maintained and bred as described previously (Shimizu et al., 2015). All euthanasia and experimental procedures are approved by the University of California Los Angeles Animal Care and Use Committee.

### Generation of MCU Mutant Line

The TALEN plasmids were gifts from Keith Joung (TAL3112, Addgene plasmid # 41286; http://n2t.net/addgene:41286; RRID:Addgene_41286 and TAL3113, Addgene plasmid # 41287; http://n2t.net/addgene:41287; RRID:Addgene_41287; Sander et al., 2011). The TALEN plasmids were linearized by *Xmn*I and used as templates for *in vitro* transcription to synthesize capped mRNAs with the mMESSAGE mMachine T7 kit (Life Technologies). mRNAs were mixed at 1:1 ratio to a final concentration of 300 pg/nl. A total of 1 nl of TALEN mRNAs was injected into the 1–2 cell stage zebrafish embryos. To identify founders, approximately 100 injected embryos were raised to adulthood and were outcrossed to wildtype fish. F1 progeny were assayed for the presence of mutations in the *MCU* locus by PCR (primer sequences: F: 5‘- CACTTCAGAGATGGCTGCGAAAGTGTG -3′, R: 5′- CTCG GTTTCAATTCCGGGGACTCAC-3′).

### Quantitative Real-Time PCR

RNA was isolated from 3 days post fertilization embryos using TRIzol (Life Technologies) and cDNA was synthesized with the iScript cDNA Synthesis Kit (Bio-Rad). Real-time PCR was carried out using LightCycler SYBR Green reagents on a LightCycler 480 system (Roche). Primers used included (5′ to 3′):

bactin-28F: GTTGACAACGGCTCCGGTATGTG
bactin-477R: CACACCATCACCAGAGTCCATCAC
mcu-994F: AAGAGGACACGCTTTGACATTGAGAAG
mcu-1114R: CGATTTGCTGGATAGGGAGATTCAACTG
atp2b1a-F: TGTCTGTTTATCTTGCGCAATG
atp2b1a-R: AAACCATACACGATACTCGACA
cldni-F: AGTGTAAAAGCTACGACTCGTT
cldni-R: CATGCAGCTTATGATGGTCATG
kcne4-F: GCACAAATCTAAACTAACGGCT
kcne4-R: GCACTATTTCGTACGACTGAAC
kcnj5-F: CATATTGGGGTCGATTGTCAAC
kcnj5-R: CTGATTGAGCGGAATAAACTCG

Relative expression of target genes was calculated using the 2^(−ΔΔCt) method.

### Cardiomyocyte Isolation and Evaluation of Mitochondrial Ca^2 +^ Uptake

Cardiomyocytes were isolated from the ventricles of WT and *MCU* mutant hearts as previously described (Sander et al., 2013). To measure mitochondrial Ca^2 +^ uptake, cardiomyocytes were loaded with 5 μM Rhod-2/AM (Invitrogen) in minimum essential medium with 10 mmol/L BDM, 2 mM/L Glutamax, 100 U/mL penicillin, 100 μg/mL streptomycin and 5% fetal calf serum for 30 min at 4°C followed by a 30 min de-esterification at 37°C (Trollinger et al., 2000). Subsequently, cells were transferred to Ca^2 +^ free Tyrode’s solution containing 2 μM thapsigargin and platted on 35 mm glass bottom dishes (MetTak). To block mitochondrial Ca^2+^ uptake, cells were treated 10 μM Ru360 for 30 min at room temperature. Fluorescent signals at 561 nm excitation/580 nm emission were monitored using a Zeiss LSM 510 confocal microscope (Carl Zeiss, Germany) every 15 s from 1 min before to 2 min after Ca^2 +^ bolus was added (Shimizu et al., 2015). Changes of Rhod 2 fluorescence were measured by ImageJ (ΔF/F_0_).

### Histology and Immunohistochemistry

Freshly dissected adult zebrafish hearts were embedded in OCT and sectioned at 5 μm by the UCLA Translational Pathology Core Laboratory. Sections were fixed and permeabilized with acetone, then stained with anti-sarcomeric α-actinin (1:1000, clone EA-53, #A7732, Sigma-Aldrich) overnight at 4°C. Confocal images were acquired using a Zeiss LSM 510 confocal microscope (Zeiss, Germany) with a 40X oil immersion objective. Measurement of area was performed in ImageJ.

### Transmission Electron Microscopy

Dissected ventricles were fixed in 2.5% glutaraldehyde and 2% formaldehyde in 0.1 M sodium phosphate buffer (PB) overnight at 4°C. After washing, samples were then post-fixed in 1% osmium tetroxide in 0.1 M PB, and dehydrated through a graded series of ethanol concentrations. After infiltration with Eponate 12 resin, the samples were embedded in fresh Eponate 12 resin and polymerized at 60°C for 48 h. Ultrathin sections of 70 nm thickness were prepared and placed on formvar coated copper grids and stained with uranyl acetate and Reynolds’ lead citrate. The grids were examined using a JEOL 100CX transmission electron microscope at 60 kV and images were captured by an AMT digital camera (Advanced Microscopy Techniques Corporation, model XR611). Preparation of samples for electron microscopy was carried out by the UCLA Brain Research Institute Electron Microscopy Core Facility.

### *In vivo* Surface ECG Recording and Analysis

Experiment and preparation involving ECG, including tricaine concentration and electrode positioning and signal acquisition were performed as previously described (Zhao et al., 2019). In brief, experiments were conducted with needle electrodes and data acquisition hardware PowerLab3/45 by AD Instruments. Surface ECGs were approximately 2–3 min in length and were measured within 10 min after first exposure to anesthesia. All statistical analysis performed for adult fish (*n* = 28) were based on regular (distinct waveforms, but not necessarily rhythmicity) consecutive cardiac cycles for a minimum of 1 min. Atrial rate, ventricular rate, and heart rate were analyzed automatically using LabChart 8 Pro by AD Instruments. *P*-*P* intervals, the magnitude of P and R amplitudes, and the duration of QRS complexes were calculated manually for each cardiac cycle for the entire > 1 min duration (*n* = 3563, *n* = 3563, *n* = 2919, *n* = 2919, for *P*-*P* interval, *P* amplitude, *R* amplitude, and QRS complex duration, respectively). Additional rhythmicity analysis was performed by screening *P*-*P* intervals greater than 1.0 s. Statistical analysis performed using an unpaired student *t*-test.

### Image Analysis on Ventricular to Atrial Planar Surface Area Ratio

High resolution video recordings of dissected adult zebrafish heart, positioned in a frontal planar orientation, were analyzed by processing individual frames through imaging analysis ImageJ (NIH). Frames during systole and diastole were selected to calculate and average ventricular to ASA ratio.

### RNA-seq

Ten hearts from 7-month old wild type or *MCU* mutant zebrafish were pooled for each sample and RNA was extracted using TRIzol (Life Technologies) and purified with the Nucleospin RNA kit (Machery Nagel). Libraries for sequencing were prepared by the UCLA Technology Center for Genomics and Bioinformatics and sequencing was performed on an Illumina HiSeq3000 system.

FASTQ files were aligned to the Ensembl Zv9 genome reference using TopHat v2.1.0 (Kim et al., 2013) and differential expression analysis was performed using Cufflinks v2.2.1 (Ghosh and Chan, 2016). The volcano plot of differential gene expression was produced in R v3.5.1 by Galaxy (Afgan et al., 2018).

### GO Enrichment Analysis

GO annotations for zebrafish genes were retrieved from the Molecular Signatures Database v7.1 (Subramanian et al., 2005; Liberzon et al., 2011) and GO enrichment analysis was performed in R v3.6.3 using the clusterProfiler package (Yu et al., 2012). Dot and gene concept network plots were made using the enrichplot package in R v3.6.3.

## Data Availability Statement

The original contributions presented in the study are publicly available. This data can be found here: ENA (European Nucleotide Archive). Primary Accession: PRJEB40865; Secondary Accession: ERP124560.

## Ethics Statement

The animal study was reviewed and approved by UCLA Chancellor’s Animal Research Committee.

## Author Contributions

AL, HS, CK, and J-NC conceived the project and the overall design of the experimental strategy. AL, HS, WH, YZ, AB, and J-NC conducted the experiments. AL, WH, and J-NC prepared the manuscript. All authors contributed to the article and approved the submitted version.

## Conflict of Interest

The authors declare that the research was conducted in the absence of any commercial or financial relationships that could be construed as a potential conflict of interest.

## Supplementary Material

Supplementary Figure 1 Quantitative PCR analysis of expression of selected genes in adult WT and *MCU* mutant hearts.

Supplementary Movie 1 Explanted zebrafish heart.

Supplementary Movie 2 Explanted zebrafish *MCU* mutant heart.
