# Supplementary figures and images for: Mitochondrial Calcium Uniporter Deficiency in Zebrafish Causes Cardiomyopathy With Arrhythmia
Source: Front Physiol. 2020 Dec 23;11:617492. doi: 10.3389/fphys.2020.617492 (PMC7785991; doi:10.3389/fphys.2020.617492)

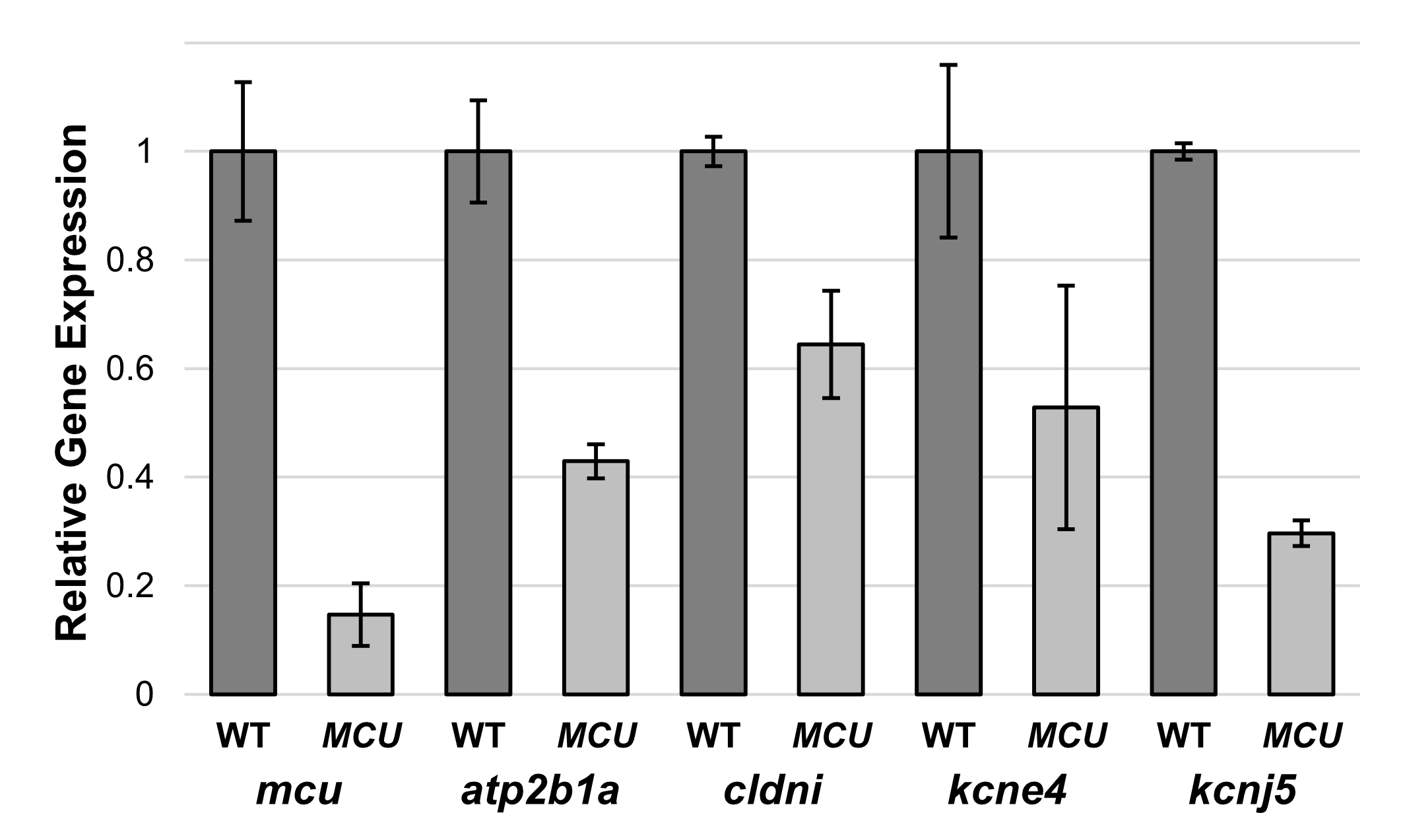

Supplement: Supplementary Figure 1 — Quantitative PCR analysis of expression of selected genes in adult WT and MCU mutant hearts. [file Image_1.TIF]
